# A CUP HALF FULL: EXPLORING THE KINEMATIC CONSEQUENCES OF VARIATIONS IN THE DRINKING TASK PROTOCOL

**DOI:** 10.2340/jrm.v57.43843

**Published:** 2025-10-21

**Authors:** Justin HUBER, Stacey SLONE, Ann M. STOWE

**Affiliations:** 1 Department of Physical Medicine and Rehabilitation, University of Kentucky, Lexington; 2 Dr. Bing Zhang Department of Statistics, University of Kentucky, Lexington; 3 Departments of Neurology and Neuroscience, University of Kentucky, Lexington, USA

**Keywords:** activities of daily living, motor skills, biomechanics, stroke, rehabilitation, upper extremity

## Abstract

**Objective:**

Kinematic assessment of the drinking task offers objective metrics of upper limb recovery following neurological injury. The rehabilitation research community’s increased interest has led to consensus standardization efforts. These efforts inherently depend on fidelity of the activity protocol underlying drinking task kinematics. This study’s objective is to investigate whether differences in the drinking task protocol, as observed in prior literature, impact common kinematic metrics.

**Design:**

Incomplete block design with repeated-measures.

**Subjects/Patients:**

Six neurotypical participants.

**Methods:**

Seating position, cup start position, and target definition for cup return were varied. Mixed effects linear models analysed the impact of protocol variants on validated kinematic metrics used in stroke rehabilitation research.

**Results:**

All considered factors have a significant impact on at least 1 kinematic metric. Seating position impacts movement time (*p* = 0.035) and trunk displacement (*p* = 0.017), cup starting position impacts trunk displacement (*p* = 0.001), and target definition impacts movement time (*p* = 0.036). Of note, none of the factors significantly altered the number of movement units.

**Conclusion:**

Further refinement and adherence to a standardized protocol for the drinking task activity may reduce between-study effects and promote the successful uptake of upper limb kinematic assessment in the rehabilitation research community.

## LAY ABSTRACT

Movement of the upper limb is commonly impaired after neurological injuries such as stroke. Rehabilitation is a cycle that relies on accurate assessment to guide treatments. Kinematic assessment of the upper limb involves measuring motion of the human body during movement. Kinematics of the drinking task have been recommended for use in the rehabilitation research community. However, performance of a task may depend on how the task is set up. Among prior studies, despite detailed descriptions of the drinking task setup, differences persist and their impact on kinematics is unclear. This pilot study explored how slight variations in the drinking task setup might impact kinematic assessment among 6 adults with typical neurological function. We found that every variation led to significant changes in at least 1 kinematic outcome. This suggests a need for continued refinement of the drinking task protocol to improve repeatability and to support uptake.

Recent longitudinal surveys of stroke survivors reveal that motor recovery of the upper limb is just as important as recovery of walking (1). Upper limb movement is impaired in over 80% of individuals experiencing stroke (2). Over the past 20 years, kinematic assessment of the upper limb has garnered increased attention among rehabilitation researchers. This can be attributed to an ongoing desire among clinical stakeholders for more objective and granular metrics to assess the recovery trajectories of patients, to guide patient expectations, and to guide allocation of limited rehabilitation resources. Within the stroke rehabilitation research community, an international panel has recommended standardized use of upper limb kinematic metrics (3). While these recommendations suggest a critical mass, the successful adoption of upper limb kinematic metrics in the field will additionally depend on reproducibility of activity protocols behind the metrics.

Numerous studies of upper limb kinematics have included the drinking task activity protocol as performed by a broad age range of neurotypical adult and paediatric populations (4–14). Importantly, for rehabilitation providers, these studies have also captured critical data related to pathological conditions such as musculoskeletal (15, 16) and neurological disease (17–23). The appeal of the drinking task has been attributed to its movement complexity, its goal-oriented task specificity, and its cyclical motion (24). Despite a plethora of researchers interested in the drinking task, prior studies feature nuances in the drinking task activity protocol. These variations range from seemingly minor (e.g., participant seating on a stool vs chair with seatback) to more obvious (e.g., participant standing vs sitting). These variations underscore the challenge of standardizing the drinking task, which is an activity that can occur in numerous possible environments and can concur with other tasks in daily life (25). In recent years, these challenges have motivated studies with ever-increasing rigour (26) – a much-needed trend to enhance the value of drinking task kinematics.

This pilot study investigated a protocol for the drinking task activity, which has been previously developed for kinematic assessment of the post-stroke upper limb (26). The protocol was replicated in accordance with published methods while being mindful of factors described with nuanced differences in the literature. An incomplete randomized block experiment was then used to analyse how 3 factors, when implemented differently in the activity protocol, subsequently impact kinematic outcomes. Specific kinematic outcome metrics were selected that have been shown to be valid, reliable, and responsive to clinical change in stroke rehabilitation research – namely, (*i*) trunk displacement (TD), (*ii*) movement time (MT), and (*iii*) number of movements units (NMU) (5, 17–19). The hypothesis was that variations in the 3 factors significantly impact kinematic metrics of the drinking task in a cohort of healthy, neurotypical adults.

## METHODS

### Participants

A total of 6 neurotypical adults were recruited from a sample of convenience. Self-reported demographics and participant characteristics are summarized in Table I. For arm length of the dominant upper limb, the limb was positioned in neutral position at the side with shoulder adducted and elbow extended, and distance was measured from acromion to ulnar styloid. Exclusion criteria were any condition affecting motor abilities of the upper limb. Each participant presented to an established research lab located at a freestanding inpatient rehabilitation hospital. Participants performed 4 repetitions of the drinking task with their dominant upper limb (27), and each repetition comprised a unique variation of protocol factors.

**Table I:** Participant demographics

Participant	Gender/sex	Age (years)	Arm length (cm)	Handedness
P1	F	35	55.95	R
P2	M	61	60.00	R
P3	M	27	61.95	R
P4	M	37	63.90	R
P5	F	33	52.95	R
P6	F	35	56.40	R

M: male; F: female; R: right.

The study was approved by the University of Kentucky Institutional Review Board (IRB #63176, Ethical Committee #423), Lexington, KY, USA), and informed written consent was obtained from all participants prior to data collection.

### Study design

For this pilot study of the drinking task activity protocol, an incomplete randomized block experiment was implemented to investigate multiple factors while limiting the number of trials per participant. The drinking task activity requires an individual to perform 5 different phases of movement including reaching to grasp cup, forward transport of cup to mouth, drinking, back transport, and return to starting position (26). Throughout the literature, protocols for the drinking task activity have considered a wide variety of factors including, for example, the dimensions of the cup, the volume of water, and the tabletop height.

**Fig. 1:**
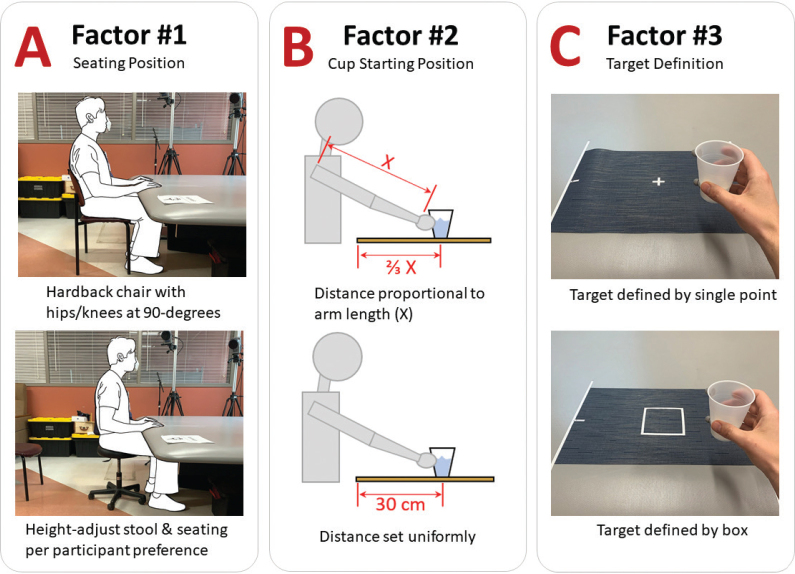
Factors of interest in drinking task activity. Three factors were investigated including seating position (panel A), cup starting position (panel B), and target definition for cup return (panel C). Each factor was varied according to 2 defined factor levels.

A limited subset of factors was selected that conceivably might impact the kinematic metrics of interest (i.e., TD, MT, NMU). The following factors were chosen: (*i*) seating position, (*ii*) cup start location, and (*ii*) target definition for cup return (Fig. 1). Two levels were defined for each of these factors. In past studies, the seating position has been described with regard to the chair seatback and with regard to the joint angles of the participant’s hips and knees (15, 21, 22, 26). To investigate seating position, participants were instructed to either (a) use a chair with seatback and ensure hip and knees flexed 90 degrees or (b) use a stool and assume a seating position as per their comfort without specific guidance on joint position. With regard to the cup’s starting location, past studies have described a location at a fixed distance (26, 28, 29) or at a distance proportional to the participant’s arm anatomy (6, 9, 22). To investigate this aspect of the protocol, the cup’s starting position was defined at either (a) a fixed distance of 30 cm away from the table edge or (b) a distance equal to 2/3 the participant’s measured arm length. With regard to the target definition for returning the cup, past studies have described lines, boxes, or no targets at all (4, 5, 8, 21, 26). To investigate this factor, the target was defined as either (a) a single point located centrally to the cup’s start position or (b) a 10 cm by 10 cm box with centroid located 30 cm away from the table edge.

To collect kinematic metrics of the drinking task, a marker-based motion capture system was used (Oqus 100, Qualisys AB, Gothenburg, Sweden). This system comprises 5 optoelectronic cameras recording at 240 Hz and with infrared sensors to capture reflective markers applied to the participants. The marker setup included 10 reflective markers placed on landmarks of the participant’s limbs and thorax as well as 2 reflective markers on the cup (26). From distal to proximal, the landmarks were as follows: 3rd metacarpophalangeal joint (bilateral), ulnar styloid (bilateral), lateral epicondyle (bilateral), midway of the acromion (bilateral), sternal notch, and midline between the eyes in line with the supraorbital ridge.

### Data processing

**Fig. 2:**
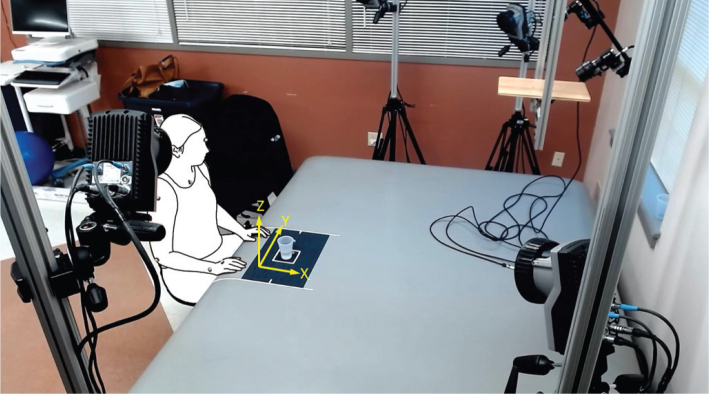
Motion capture setup. To measure kinematics of the drinking task activity, 5 optoelectronic cameras measured motion of skin-adhered markers relative to the depicted origin and cardinal plane.

The raw data from the marker-based motion capture system consisted of a time-series of approximated joint positions in 3-dimensional space. The motion capture setup was tailored to an activity space with defined origin and cardinal planes (Fig. 2). The joint position data were subsequently post-processed in MATLAB (MATLAB 2024b, Mathworks Inc., Natick, MA, USA) using a 2nd order, zero-phase lag Butterworth low-pass filter with 6Hz cutoff frequency (19, 26). Subsequently, the drinking task kinematic metrics (TD, MT, NMU) were computed with MATLAB routines consistent with prior literature (26). The operationalized definitions for each metric are listed in Table II.

**Table II:** Definitions of kinematic metrics

Kinematic metric of drinking task	Definition
Trunk displacement, TD (mm)	Maximum displacement of the sternal reflective marker during the drinking task as compared with the marker’s initial position
Movement time, MT (sec)	Duration of drinking task based on start/stop times defined as when hand velocity exceeds or falls below 2% of peak velocity, respectively
Number of movement units, NMU (count)	Movement unit defined as a local minimum in the hand velocity profile followed by a local maximum that exceeds amplitude of 20 mm/s, and time between 2 subsequent peaks is ≥150 ms. For drinking task, the minimum value for NMU is 4 (1 movement unit per movement phase)

### Statistical analysis

Using kinematic metrics as the dependent outcomes, a mixed-effects linear model was applied to investigate the effect of and variation within each factor. For dependent outcomes with non-normal distributions (i.e., TD), logarithm transformations were applied (TD_log_). For TD, 1 outlier was noted that even the log-transform did not stabilize. Models were run with and without the outlier to assess robustness of the conclusions. As the removal of the outlier did not change the conclusions, the reported results include the outlier. Given the exploratory aim of the study, subject and repetition were considered as random effects in all the statistical models. Statistical significance was defined by *p*-values less than 0.05, and no adjustment for multiple comparisons was implemented due to the pilot nature of this study. For factors associated with significant changes in a dependent outcome, additional box-and-whisker plots were used to visually compare variance of the dependent outcome in respect of the 2 levels defined for the factor.

## RESULTS

**Fig. 3:**
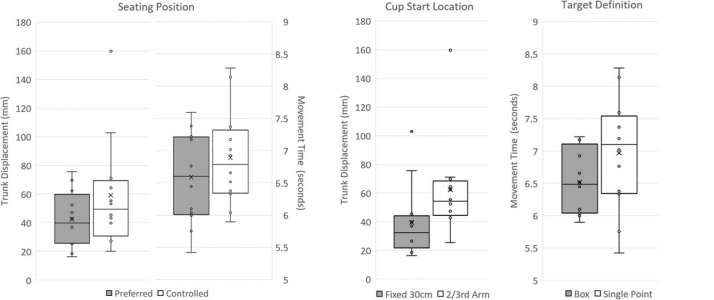
Box-and-whiskers plots. For each factor associated with a significant effect, the variance of the kinematic outcome is visualized for both factor levels.

All considered factors of the drinking task (i.e., seating position, cup starting location, and target definition) have a significant impact on at least 1 kinematic metric of the upper limb (i.e., TD and/or MT). Notably, NMU was not significantly different for any of the considered factors of the drinking task. For seating position, significant differences existed for both MT (*p* = 0.035) and TD_log_ (*p* = 0.017). Trials involving a preferred seating position with a stool resulted in a mean MT of 6.6 s (95% CI: 6.3–6.8 s) and a geometric mean TD of 37.9 mm (95% CI: 32.4–44.3mm). In comparison, trials involving controlled joint angles and a seatback chair resulted in a higher mean MT of 6.9 s (95% CI: 6.7–7.2 s) and a higher geometric mean TD of 50.7mm (95% CI: 43.4–59.3 mm. For cup starting location, a significant difference existed for TD_log_ (*p* = 0.001). Trials with the cup located at fixed distance resulted in a geometric mean TD of 34.7mm (95% CI: 29.7–40.5 mm), and trials with the cup located at a proportional distance resulted in a higher geometric mean TD of 55.5mm (95% CI: 47.5–64.8 mm). For target definition, a significant difference existed for MT (*p* = 0.036). Compared with trials with a single point target, the trials with a box target resulted in a mean MT of 6.6 s (95% CI: 6.3–6.8 s), and trials with a single point target resulted in a higher mean MT of 6.9 s (95% CI: 6.7–7.1 s). Otherwise, when comparing between factor levels, no other significant differences were found in kinematic metrics, and no significant differences were found for first-order factor interactions. For each significant relationship, the influence of factor levels on variance in the kinematic metric was visualized (Fig. 3).

## DISCUSSION

In this pilot study involving neurotypical participants, variations were introduced in an activity protocol for the drinking task, and an investigation was performed to understand the impact of these variations on 3 kinematic metrics previously developed for assessment of post-stroke motor recovery. The objective was to explore nuances of the activity protocol so as to inform standardization and implementation of kinematics of the drinking task activity in stroke rehabilitation research. Based on an incomplete randomized block experiment, the primary findings indicate that every factor has a significant impact on at least 1 kinematic metric, which underscores the need for further refinement and adherence to a standardized drinking task activity protocol.

### Seating position

Among the factors included in this pilot study, seating position may be the most important to consider when implementing the drinking task activity protocol. Prior studies have varied in their approach to specifying seating position for the drinking task. Among the more stringent approaches, researchers have specified features of the seat such as chairbacks and armrests (15, 21, 22), as well as initial participant positioning such as joint angles and hand placement (17–19, 23, 26), and even restraints in some cases (6). Conversely, other studies provide more minimal descriptions of seating and participant positioning (8).

In the present study, variations in seating position significantly impacted trunk displacement. Trunk displacement has been described as a kinematic metric of compensatory behaviour that increases as one’s ability to extend the reaching arm is impaired (17). Several aspects of seating position could impair this ability. For example, when comfortably seated without concern for initial shoulder/elbow positioning, participants could simply move their initial torso location closer to the table to reduce the horizontal reach distance. Sitting posture may also have contributed. Past studies show that different sitting postures (such as slouched vs upright seating) significantly impact scapular kinematics during arm abduction and elevation (30, 31). Similarly, the differing seating conditions in our study (seatback chair vs stool) may also impact scapular movement and thus reaching ability during the drinking task. Finally, seat height may also impact one’s ability to reach, as a higher seat height, when paired with a fixed table height, would increase downward reaching, reduce horizontal reaching capacity, and increase the compensatory lean of the trunk during the drinking activity. While seat height was not explicitly controlled, a systematic difference in seat height may have existed. For example, a comfortable seating position has been associated with seat heights less than the popliteal height to prevent thigh pressure and blood flow restriction (32, 33). In comparison, sitting with knees/hips at right angles would place the seat height at the popliteal height.

Seating position significantly impacted another kinematic metric of the drinking task: movement time. This could be simply explained by a difference in horizontal reach distance, which has a linear relationship to movement time based on empirical observation and energetics modelling (34). This could also be explained by initial core muscle activation for the drinking task. Core stabilization (and preparatory truncal motion) is known to precede movement of the upper limb to ensure spinal alignment and energy transfer (35). However, paraspinal activity has been shown to be reduced when sitting with a seatback (36). Thus, the seatback condition may necessitate added time for core activation, resulting in a longer overall movement time.

### Cup start location and target definition for cup return

Cup starting location has been described essentially in 2 ways: (a) location in proportion to the subject’s arm length (6, 9, 22) or (b) a simplified fixed distance (26). In the present study, these variations in cup starting location significantly impacted trunk displacement. This is likely explained by the difference in the reach distance. For the arm lengths of our participants (see Table 1), the proportional distance was always greater than the fixed distance, and, as expected, this greater distance resulted in higher trunk displacement. Of note, the definition of the proportional distance creates yet more opportunity for nuance. For example, some studies define a percentage of the acromion-to-index distance during maximum passive elbow extension (6) while others define a percentage of the arm’s maximum reach distance (37). When considering the stroke population, the nuances of these definitions may be further complicated by common post-stroke motor sequelae, i.e., spasticity that limits passive joint motion and synergy patterns that limit reach distance.

Return target for the cup has been described in a number of ways. Strategies include square markings (5), circumferential cup outlines (8), linear thresholds on the table (17–19, 26), or no markings (4, 21). In the present study, a variation in the target definition resulted in significant differences in movement time of the drinking task. Specifically, the single-point target resulted in a higher mean movement time compared with the square target. This may be explained by the higher accuracy in movement that is required when returning the cup to the single-point target. According to the speed–accuracy trade-off as described in Fitts’s Law (38, 39), this enhanced accuracy comes with an associated cost: increased duration of the movement. As explained through the lens of human motor planning theory, movement duration is selected to minimize endpoint variability of goal-directed arm movement (40). In the current study, a more forgiving target allows more endpoint variability, which may lead to selection of a shorter movement time.

### Variance in kinematics metrics

Variance in kinematic data appears to be consistent between levels for each factor. This suggests that the choice of factor level is relatively unimportant as long as it is implemented reliably. An exciting implication is that practical considerations could guide the decision. For example, if considering cup location based on a fixed distance or a proportional distance, then a fixed distance might be preferred to reduce burden to the subject and the evaluator (41).

### Limitations

Several study limitations should be noted. Incomplete block designs have the ability to test multiple factors with reduced trials at the expense of confounding for high-order interactions. Among factors, the comfortable seating position was purposely ill-defined, which limits interpretation. Measurements of subject-selected comfortable seating (after the selection occurs) may improve interpretation. Among the kinematic outcome measures considered, NMU exhibited low variability in this small sample of neurotypical participants, which likely limited analysis. Future studies will benefit from larger and more heterogeneous sample sizes that include neurodivergent individuals. Challenges are recognized in marker-based motion capture studies including skin movement artefact and inconsistent marker placement. The current study replicated a reduced marker setup designed for clinical use (26), which is advantageous for clinical translation but admittedly may accentuate these challenges. Furthermore, in accordance with this setup designed for clinical use, the current pilot study featured 4 activity trials performed by the participant with each upper limb whereas consensus statements have recommended 15 trials (3).

Importantly, the statistically significant findings in this pilot study may not correspond to clinical significance. For example, when participants assumed a seating position as per their comfort, a statistically significant increase in MT (approximately 0.3 s) is notably less than estimates of the minimal clinically importance difference (MCID) of MT in the literature (2.4 s). However, clinical significance based on an estimated MCID should be interpreted with caution for several reasons. First, this estimate is based on a 3-month study of first-time stroke survivors with mild/moderate impairment, which may not generalize (19). Second, this estimate is based on comparisons between kinematics and a clinician-reported outcome measure (i.e., the Action Research Arm Test, ARAT), whereas a patient-reported outcome measure may be more ideal for MCID determination (42). Last, this estimate assumes a linear relationship between changes in kinematics and changes in ARAT, which is difficult to verify given the continuous scale of the former and the ordinal scale of the latter.

### Conclusion

When assessing individuals based on the drinking task activity, variations in the activity protocol contribute to statistically significant change in kinematic metrics. The current study highlights factors, such as seating condition, that should be carefully considered as the protocol is further refined for standardized use. When identifying factors and their nuanced variations, the optimum choice may be the one that increases precision in the measured outcome. If there are no obvious differences in precision, as in the current study, then the choice may be guided by more practical considerations. For example, a fixed cup starting location may be chosen due to the practical benefit, i.e., reducing administrative burden. Overall, the growing wealth of kinematic studies on the drinking task, when combined with universal standardization of the activity protocol, not only offers value for test–retest following interventions, but also potential for comparisons between populations in various rehabilitation settings and at various geographic sites.
